# Corrigendum

**DOI:** 10.1002/jcsm.12811

**Published:** 2021-10-07

Physical‐function derived cut‐points for the diagnosis of sarcopenia and dynapenia from the Canadian Longitudinal Study on Aging.

Volume 10, Issue 5, pages: 985–999.

First published online: July 15, 2019.

In the original full paper,^1^ appendicular lean mass data obtained from the CLSA inadvertently included bone mineral content. Because sarcopenia is typically defined by low appendicular soft lean mass (without bone), cut‐points to identify sarcopenia were overestimated. Bone mineral content data were subsequently obtained from the CLSA and subtracted from lean mass for correction; all original analyses were repeated.

Correct appendicular (soft) lean mass and index values are found in Table 1. Cut‐points for low appendicular (soft) lean mass are 7.31 kg/m^2^ in men and 5.43 kg/m^2^ in women (Figure 3). This correction impacted mostly descriptive data by sarcopenia category and estimations of sarcopenia prevalence in this cohort (Tables 2 and 3; Suppl. Figure 2), and in comparison to other cohorts (Tables 4 and 5). However, the correction did not affect the relationships between low appendicular lean soft mass, handgrip strength and physical function (Figure 1) and therefore, the original interpretation of data and conclusions remain.

**Table 1:** Baseline characteristics of the Canadian longitudinal study on aging participants by sex, 2011–2015

	Men (*n* = 4,725)	Women (*n* = 4,363)
Age, year	72.7 ± 5.5	72.5 ± 5.5
Caucasian, %	96.1	97.5
Anthropomorphic measurements height, cm	1.74 ± 0.07	1.60 ± 0.06
Weight, kg	83.9 ± 13.5	70.1 ± 13.5
BMI, kg/m^2^	27.8 ± 4.0	27.5 ± 5.1
Current smoker, %	5	5
Nutritional risk (SCREEN II‐AB; 0–48)	39.6 ± 5.5	39.0 ± 5.9
Medication number (range 0–11)	0.8 ± 0.9	1.0 ± 1.0
PASE score (range 0–629)	129 ± 59	111 ± 53
Body composition		
ALM, kg	24.36 ± 3.59	16.23 ± 2.74
ALM index, kg/m^2^	8.05 ± 0.99	6.34 ± 0.95
Fat mass, kg	25.02 ± 7.59	29.01 ± 8.89
Strength		
Maximum grip strength, kg	39.8 ± 8.4	23.9 ± 5.1
Physical performance		
BMI‐adjusted physical performance, *Z* score	0.17 ± 2.14	−0.18 ± 2.16
TUG, s	9.9 ± 1.9	10.0 ± 2.0
Gait speed, m/s	0.95 ± 0.19	0.92 ± 0.18
Balance (range 0–60 s)	28.6 ± 23.1	25.1 ± 22.3
Chair rise average time, s	2.8 ± 0.8	2.9 ± 0.8

Values are mean ± SD. ALM, appendicular lean mass; BMI, body mass index; PASE, Physical Activity Scale for Elderly; SCREEN II, Seniors in the Community Risk Evaluation for Eating and Nutrition; TUG, timed‐up‐and‐go.

**Figure 3:**
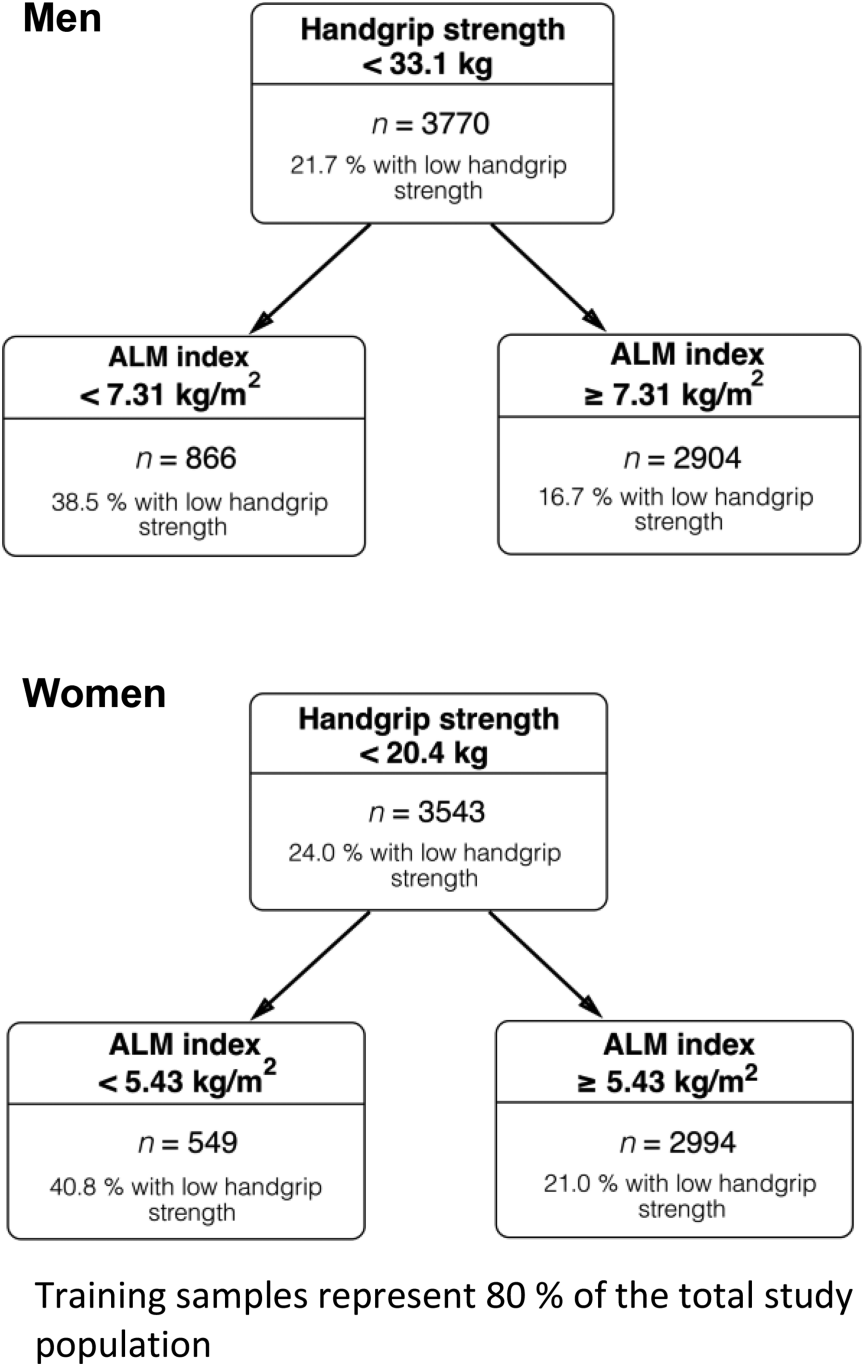
CART results from training samples illustrating the ALM index cut‐points as predictors of low handgrip strength in men and women.

**Table 2:**
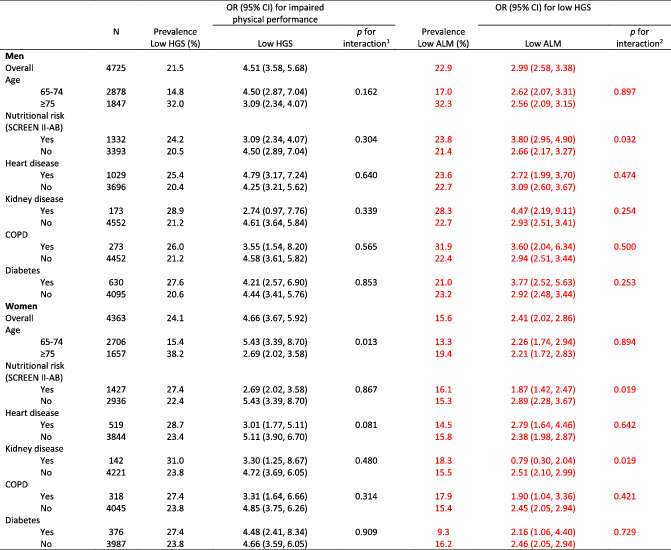
Sensitivity analysis for strength as a predictor of limited physical performance and for low ALM as a predictor of low strength across subgroups in the CLSA cohort, 2011–2015

HGS, handgrip strength; ALMI, appendicular lean mass index; SCREEN II‐AB, abbreviated Seniors in the community risk evaluation for eating and nutrition, version II, score < 38 was considered as at risk of poor nutritional state; COPD, chronic obstructive pulmonary diseases.

^1^ Interaction for absence/presence of low HGS and subgroup characteristics in the prediction of impaired physical performance.

^2^ Interaction for absence/presence of low ALM and subgroup characteristics in the prediction of low HGS.

**Table 3:**
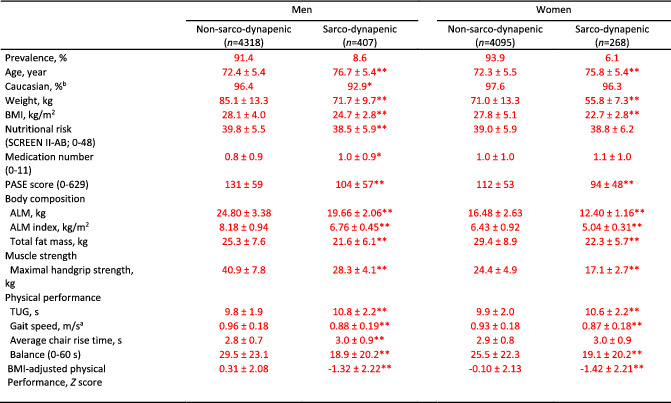
Baseline characteristics of men and women by absence or presence of sarco‐dynapenia applying Canadian longitudinal study on aging cut‐points, 2011–2015

Values are mean ± SD. ALM, appendicular lean mass; BMI, body mass index; PASE, Physical Activity Scale for Elderly; SCREEN II‐AB, abbreviated Seniors in the Community Risk Evaluation for Eating and Nutrition, version II; TUG, timed‐up‐and‐go. Mann–Whitney *U* test unless otherwise specified.

^a^ Independent *t*‐test;

^b^ Chi‐square test

* *P*‐value < 0.05;

** *P*‐value < 0.001;

**Table 4:**
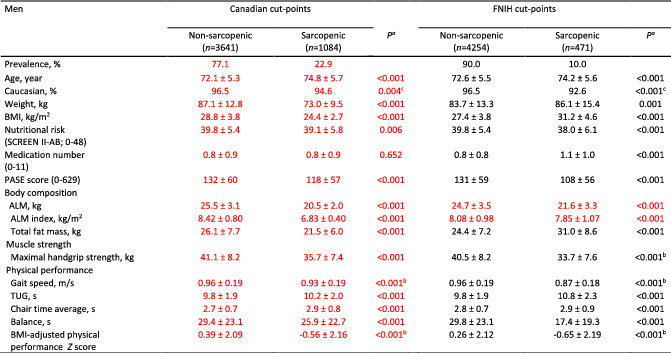
Descriptive statistics between men with presence or absence of low ALM applying the new Canadian and the FNIH cut‐points, in the Canadian longitudinal study on aging cohort

Values are mean ± SD. ALM, appendicular lean mass; BMI, body mass index; FNIH, Foundation for the National Institute of Health; PASE, Physical Activity Scale for Elderly; SCREEN II‐AB, abbreviated Seniors in the Community Risk Evaluation for Eating and Nutrition, version II; TUG, timed‐up‐and‐go.

^a^ From Mann–Whitney *U* test unless otherwise specified; ^b^ Independent t‐test; ^c^ Chi‐square test.

**Table 5:**
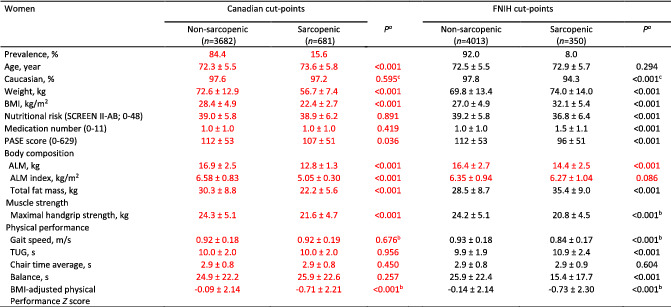
Descriptive statistics between women with presence or absence of low ALM applying the new Canadian and the FNIH cut‐points, in the Canadian longitudinal study on aging cohort

Values are mean ± SD. ALM, appendicular lean mass; BMI, body mass index; FNIH, Foundation for the National Institute of Health; PASE, Physical Activity Scale for Elderly; SCREEN II‐AB, abbreviated Seniors in the Community Risk Evaluation for Eating and Nutrition, version II; TUG, timed up‐and‐go.

^a^ From Mann–Whitney *U* test unless otherwise specified.

^b^ Independent t‐test.

^c^ Chi‐square test.

Corrected data are identified in red font in Tables 1, 2, 3, 4, 5 below, Figure 3, Supplemental Figure 2 and in the article text:

## Supporting information


**Table S1.** Agreement of low handgrip strength cut‐points with impaired physical performance.
**Table S2**. Agreement of low lean mass cut‐points with low handgrip strength.
**Table S3**. Agreement of the CLSA with the FNIH criteria for sarcopenia (low lean mass).
**Table S4**. Agreement of the CLSA with the FNIH criteria for sarco‐dynapenia.
**Figure S2**. Prevalence rates of impaired physical performance, low strength and low lean mass.
