# Supplementary material for: Corrigendum
Source: J Cachexia Sarcopenia Muscle. 2021 Oct 7;12(6):2262–7. doi: 10.1002/jcsm.12811 (PMC8718065; doi:10.1002/jcsm.12811)
Supplement: Supplementary file 1 — Table S1. Agreement of low handgrip strength cut‐points with impaired physical performance. Table S2. Agreement of low lean mass cut‐points with low handgrip strength. Table S3. Agreement of the CLSA with the FNIH criteria for sarcopenia (low lean mass). Table S4. Agreement of the CLSA with the FNIH criteria for sarco‐dynapenia. Figure S2. Prevalence rates of impaired physical performance, low strength and low lean mass. [file JCSM-12-2262-s001.docx]

**Supplementary Table 1**. Agreement of low handgrip strength cut-points with impaired physical performance

|  | **Men** | **Women** |
| --- | --- | --- |
| Positive percent agreement, % | 16.5 | 16.1 |
| Negative percent agreement, % | 95.8 | 96.1 |
| Cohen’s kappa | 0.164 | 0.161 |

**Supplementary Table 2**. Agreement of low lean mass cut-points with low handgrip strength

|  | **Men** | **Women** |
| --- | --- | --- |
| Positive percent agreement, % | 40.1 | 25.5 |
| Negative percent agreement, % | 81.7 | 87.5 |
| Cohen’s kappa | 0.213 | 0.148 |

**Supplementary Table 3**. Agreement of the CLSA with the FNIH criteria for sarcopenia (low lean mass)

|  | **Men** | **Women** |
| --- | --- | --- |
| Positive percent agreement, % | 34.8 | 22.9 |
| Negative percent agreement, % | 78.4 | 85.0 |
| Cohen’s kappa | 0.084 | 0.055 |

**Supplementary Table 4**. Agreement of the CLSA with the FNIH criteria for sarco-dynapenia

|  | **Men** | **Women** |
| --- | --- | --- |
| Positive percent agreement, % | 50.8 | 28.8 |
| Negative percent agreement, % | 92.0 | 94.1 |
| Cohen’s kappa | 0.120 | 0.075 |

**Supplementary Figure 2. Prevalence rates of impaired physical performance, low strength and low lean mass**

**Low lean mass**

**Impaired physical performance**

**1.6%**

**Low handgrip strength**

**5.8%**

**19.4%**

**22.7%**

**6.9%**

**0.8%**

**11.2%**

**13.2%**

**2.1%**

**2.4%**

***Sarco-dynapenia***

**7.4%**
